# A bispecific antibody agonist of the IL-2 heterodimeric receptor preferentially promotes in vivo expansion of CD8 and NK cells

**DOI:** 10.1038/s41598-021-90096-8

**Published:** 2021-05-19

**Authors:** Katherine E. Harris, Kyle J. Lorentsen, Harbani K. Malik-Chaudhry, Kaitlyn Loughlin, Harish Medlari Basappa, Sharon Hartstein, Ghenima Ahmil, Nicole S. Allen, Brian C. Avanzino, Aarti Balasubramani, Andrew A. Boudreau, Karen Chang, Maria-Cristina Cuturi, Laura M. Davison, Dennis M. Ho, Suhasini Iyer, Udaya S. Rangaswamy, Preethi Sankaran, Ute Schellenberger, Roland Buelow, Nathan D. Trinklein

**Affiliations:** 1 Teneobio, Inc., Newark, CA USA; 2 Inserm, Centre de Recherche en Transplantation Et Immunologie, UMR 1064, Nantes Université, 44000 Nantes, France

**Keywords:** Antibody therapy, Cancer therapy

## Abstract

The use of recombinant interleukin-2 (IL-2) as a therapeutic protein has been limited by significant toxicities despite its demonstrated ability to induce durable tumor-regression in cancer patients. The adverse events and limited efficacy of IL-2 treatment are due to the preferential binding of IL-2 to cells that express the high-affinity, trimeric receptor, IL-2Rαβγ such as endothelial cells and T-regulatory cells, respectively. Here, we describe a novel bispecific heavy-chain only antibody which binds to and activates signaling through the heterodimeric IL-2Rβγ receptor complex that is expressed on resting T-cells and NK cells. By avoiding binding to IL-2Rα, this molecule circumvents the preferential T-reg activation of native IL-2, while maintaining the robust stimulatory effects on T-cells and NK-cells in vitro. In vivo studies in both mice and cynomolgus monkeys confirm the molecule’s in vivo biological activity, extended pharmacodynamics due to the Fc portion of the molecule, and enhanced safety profile. Together, these results demonstrate that the bispecific antibody is a safe and effective IL-2R agonist that harnesses the benefits of the IL-2 signaling pathway as a potential anti-cancer therapy.

## Introduction

Engaging the immune system in the fight against cancer has been firmly established, with Interleukin-2 (IL-2) being one of the first recombinant proteins to be successfully used as a treatment for cancer nearly 40 years ago^1,2^. IL-2 is a key regulator of immune cells inducing both T-cell and natural killer (NK) cell proliferation. However, IL-2 is a pleiotropic cytokine that also induces the proliferation of immunosuppressive regulatory T (T-reg) cells^3^. The different functions of IL-2 are determined by the composition of IL-2 receptor complex subunits expressed on different target cells^4^. The high affinity IL-2 receptor complex is composed of IL-2Rα (CD25), IL-2Rβ (CD122), and the common gamma chain receptor IL-2Rγ (CD132) and is expressed constitutively on CD4^+^ FoxP3^+^ T-reg cells and transiently on activated T-cells^5^. The intermediate affinity receptor is composed of IL-2Rβ and IL-2Rγ and is expressed on resting T-cells, CD8^+^ effector memory T-cells, and NK-cells^5,6^. The IL-2Rα subunit is not required for downstream JAK-STAT signaling, but its association with IL-2Rβ and IL-2Rγ provides a 100-fold higher affinity to IL-2 compared to the heterodimeric receptor composed only of IL-2Rβ and IL-2Rγ^6^. Based on these receptor binding differences and cell-specific expression, it has been proposed that immunosuppressive T-regs serve as a buffer to consume low levels of IL-2 and create a threshold effect for IL-2-mediated expansion of effector lymphocytes^7^.

Because of its unique signaling properties, low dose rhIL-2 has been used in the clinic to stimulate T-regs to treat autoimmunity, while high-dose rhIL-2 (Proleukin®) was developed and approved for the treatment of metastatic melanoma and metastatic renal cell carcinoma, with durable responses in 7–12% of patients^8–13^. However, its short half-life and narrow therapeutic window have created significant challenges for the safe and effective use of rhIL-2 in patients. Specifically, rhIL-2 has severe side effects including vascular leak syndrome, hypotension, and liver toxicities that have limited its use in cancer immunotherapy. It has been shown that the vascular leak toxicity is related to the expression of the high affinity IL-2Rαβγ on vascular and lung endothelial cells leading to pulmonary edema^14^. The anti-tumor effects of rhIL-2 are further compromised by its preferential binding to the high affinity receptor on T-reg cells, blunting its efficacy as an anti-cancer therapy^15,16^. As an example, in melanoma patients receiving high dose rhIL-2 therapy, costimulator-positive (ICOS+) T-reg cells were found to be the most proliferative lymphocyte population in the blood after treatment with rhIL-2 and high numbers of ICOS+ T-regs corresponded with the worst patient outcomes^17^.

Due to the pleiotropic nature of native IL-2 and its associated limitations as a therapeutic molecule, there has been substantial effort in the field to engineer rhIL-2 variants that reduce dose-limiting toxicities and thereby broaden the therapeutic window^18,19^. Variant proteins that avoid the preferential activation of high-affinity IL-2R-expressing cells such as T-regs and vascular endothelial cells is one approach to achieving this goal. In an effort to create such a molecule, groups have mutated the IL-2Rα binding interface on IL-2, attached poly-ethylene glycol to the IL-2 protein, created synthetic IL-2 proteins, and generated an antibody that blocks the IL-2Rα binding domain^20–25^. As an alternative to IL-2, other groups have engineered IL-15 variants that bind to IL-2Rβ/IL-2Rγ subunits. The IL-15 receptor alpha subunit naturally binds to IL-15 in trans from antigen presenting cells, therefore an active IL-15 recombinant protein requires a single chain construct that expresses both IL-15 and the IL-15 receptor alpha subunit^26^. Mutated cytokines have also been fused to antibodies or Fc domains to increase the in vivo half-life of the molecules and localize the cytokine to the site of the tumor^18,27,28^. While some of these engineered proteins have the desired functional activity, many of them suffer from high levels of immunogenicity in vivo and present challenges with in vivo stability and manufacturing^24,29–32^. Therefore, creating an anti-tumor agonist of the IL-2 pathway with the desired biological activity, safety profile, and ideal drug-like properties remains an unmet need in the field.

We took a novel approach that combines the favorable drug-like properties of antibodies with the functional behavior of a molecule that facilitates IL-2Rβ and IL-2Rγ association and downstream signaling. With these criteria in mind, we created a panel of fully human bispecific antibodies that simultaneously bind IL-2Rβ and IL-2Rγ subunits and therefore mimic the activity of IL-2 while avoiding binding to IL-2Rα. To create these bispecific antibodies, we used our unique next generation sequencing (NGS)-based antibody discovery platform using humanized rats (UniRats) to identify a large collection of binding domains with a wide range of agonist activity^33,34^. The resulting bispecific IL-2Rβγ agonist antibodies exhibit the desired activation and expansion of immune effector cells, while also avoiding preferential expansion of suppressive T-regs both in vitro and in vivo. Moreover, the bispecific agonist antibodies are well tolerated in non-human primates with no observation of vascular leak syndrome or other toxicities.

## Results

### Discovery of novel anti-IL-2Rβ and anti-IL-2Rγ heavy chain only antibodies

Our design strategy was to create a bispecific antibody that simultaneously targets both the beta and gamma subunits of the IL-2 receptor to induce activation of IL-2R signaling in human immune effector cells without preferentially activating T-regs, shifting the balance to the activation of T-effector and NK-cells. Using our NGS-based antibody repertoire discovery approach in UniRats, which produce heavy chain only antibodies with fully human variable domains (UniAbs), we sought to identify novel, anti-IL-2Rβ and anti-IL-2Rγ monospecific UniAbs that could be combined into bispecific molecules capable of binding and activating the intermediate affinity IL-2Rβγ receptor^33^.

**Figure 1:**
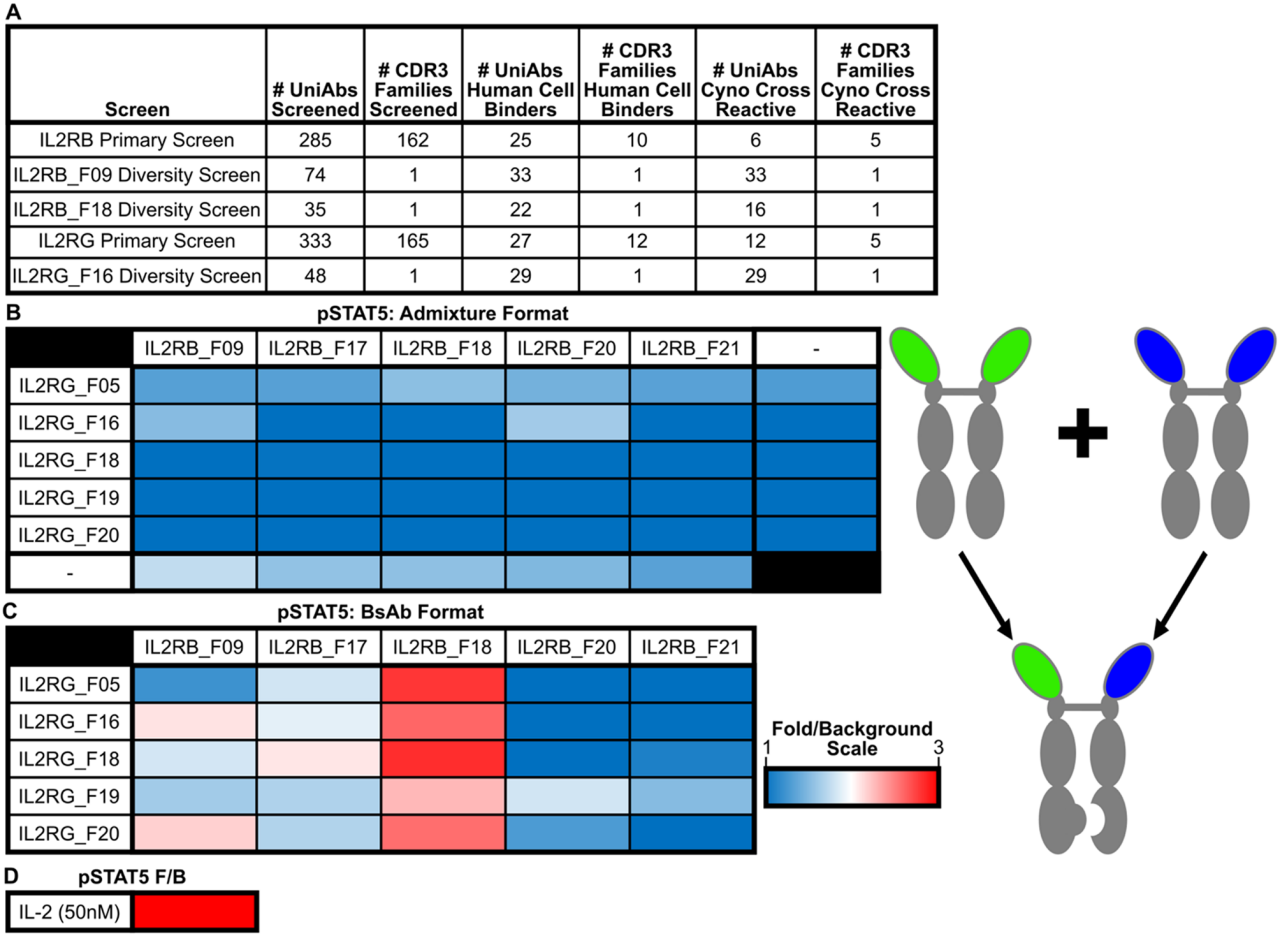
Bispecific antibodies specific to IL-2Rβ and IL-2Rγ induce phosphorylation of STAT5. (**A**) Summary of IL-2RB and IL-2RG monospecific UniAb binders identified during antibody lead discovery. (**B**,**C**,**D**) Heatmaps showing fold-induction of phosphorylated STAT5 (pSTAT5) in CD8^+^ T-cells from human PBMCs treated with individual monospecific UniAbs or αIL2Rβ and αIL2Rγ monospecific UniAbs in a 1:1 molar ratio mixture (**B**), αIL-2Rβ/γ bispecific UniAbs (**C**), or rhIL-2 positive control (**D**) at 50 nM for 1 h. In the antibody graphic on the right, the green and blue arms represent IL2RG and IL2RB binding domains, respectively. pSTAT5 levels were determined by flow cytometry and reported as geometric mean fluorescent intensity (gMFI) over the gMFI of unstimulated cells.

Applying this sequence-based discovery approach to IL-2Rβ-immunized UniRats, 285 unique heavy chain only antibodies (representing 162 unique CDR3 clonotype families) were selected for high-throughput gene assembly, recombinant expression, and primary functional screening. The resulting UniAbs were assayed for binding to recombinant human IL-2Rβ protein by enzyme-linked immunosorbent assay (ELISA) and human IL-2Rβ expressing CHO cells by flow cytometry. Cross-reactivity to cynomolgus IL-2Rβ was also assessed by binding to recombinant cynomolgus IL-2Rβ protein by ELISA as well as binding to cynomolgus IL-2Rβ expressing CHO cells by flow cytometry. Upon completion of the primary screen, 6 UniAbs (representing 5 CDR3 families) were identified that recognized both human and cynomolgus IL-2Rβ expressing cells (Fig. 1A).

Similarly, UniRats were immunized with IL-2Rγ and after NGS analysis, 333 unique UniAbs (representing 165 unique CDR3 clonotype families) were selected for high-throughput gene assembly, recombinant expression, and primary functional screening. Following expression, the resulting UniAbs were assayed for binding to recombinant human IL-2Rγ protein by Octet off-rate analysis and human IL-2Rγ expressing M07e cells using flow cytometry. Cross-reactivity to cynomolgus IL-2Rγ was assessed by binding to HSC-F cells by flow cytometry. At the conclusion of the primary screen, 12 UniAbs (representing 5 CDR3 families) were identified that recognized both human and cynomolgus IL-2Rγ (Fig. 1A).

### Identification of IL-2Rβγ bispecific antibody combinations with agonist activity

The activation of the IL-2 receptor complex triggers a signaling cascade that results in the phosphorylation of STAT5 (pSTAT5), translocation of pSTAT5 dimers to the nucleus, and transcription of STAT5-regulated genes^35,36^. As a primary assay to determine if bispecific antibodies targeting the beta and gamma subunits of IL-2R could induce activation of IL-2R signaling, 5 anti-IL-2Rβ binding arms from unique CDR3 families and 5 anti-IL-2Rγ binding arms from unique CDR3 families were combined to make 25 bispecific UniAbs for conducting an all-by-all screen of agonist activity. The bispecific UniAbs were expressed on a silenced and stabilized human IgG4 Fc (CH1 domain deleted) using knobs-into-holes technology to facilitate heavy-chain heterodimer formation, with a single anti-IL-2Rγ VH on the knob arm and a single anti-IL-2Rβ VH on the hole arm (Fig. 1)^37–42^.

A phospho-flow cytometry assay was used to measure and compare the phosphorylation of STAT5 by the 25 IL-2Rβγ UniAb combinations compared to rhIL-2 on human CD8^+^ T-cells. STAT5 phosphorylation was not observed with any of the anti-IL-2Rβ or anti-IL-2Rγ monospecific UniAbs. Similarly, STAT5 phosphorylation was also not observed when anti-IL-2Rβ and anti-IL-2Rγ monospecific UniAbs were tested as a mixture in the pSTAT5 assay, indicating that the bispecific format is necessary to bring the IL-2 β and γ receptors together to activate JAK/STAT signaling (Fig. 1B). In contrast, the bispecific UniAbs with one anti-IL-2Rβ arm and one anti-IL-2Rγ arm exhibited varying levels of agonist activity, summarized in Fig. 1C. Interestingly, the ability to induce phosphorylation of STAT5 agonist activity seemed highly dependent on the anti-IL-2Rβ arm present in the bispecific combination, while the degree of agonism appeared to be dependent on the anti-IL-2Rγ arm.

To identify antibodies with a greater range of agonist activity, a secondary diversity screen was initiated to survey other unique VH sequences in 3 of the 4 lead CDR3 clonotype families identified in the bispecific screen for STAT5 activity. These additional VH sequences were selected from the lead CDR3 clonotype families and contain sequence variation in CDR1, CDR2 and framework regions. In total, an additional 157 unique family members underwent a second round of high-throughput gene assembly, expression and were assessed for binding to IL-2R expressing cells. For IL-2Rβ, an additional 33 IL-2Rβ family 9 members and an additional 22 IL-2Rβ family 18 members that bound to human and cynomolgus IL-2Rβ cells were identified in the diversity screen. A further 29 IL-2Rγ family 16 members were identified that bound to human and cynomolgus IL-2Rγ recombinant protein and on cells in the diversity screen (Fig. 1A). This large and diverse set of novel IL-2R binding UniAbs enabled subsequent efforts to identify a set of lead IL-2Rβγ bispecific combinations with a range of functional activity.

### In vitro characterization of IL-2Rβγ bispecific UniAbs

Based on the primary and secondary binding screening results as well as the STAT5 phosphorylation seen in the all-by-all bispecific UniAb screen, 6 IL-2Rβγ bispecific UniAb molecules were selected for additional in vitro characterization. The 6 IL-2Rβγ bispecific UniAbs bound efficiently to both human and cynomolgus T-cells with a range of EC50 values (Fig. S1). Interestingly, the expression of IL-2Rβγ was approximately twofold higher on CD4^+^ T-cells than CD8^+^ T-cells across both species. None of the 6 bispecific UniAbs bound to other common gamma chain partners (IL-4R, IL-7R, IL-9R or IL-21R) or IL-2Rα by Octet off-rate analysis.

**Figure 2:**
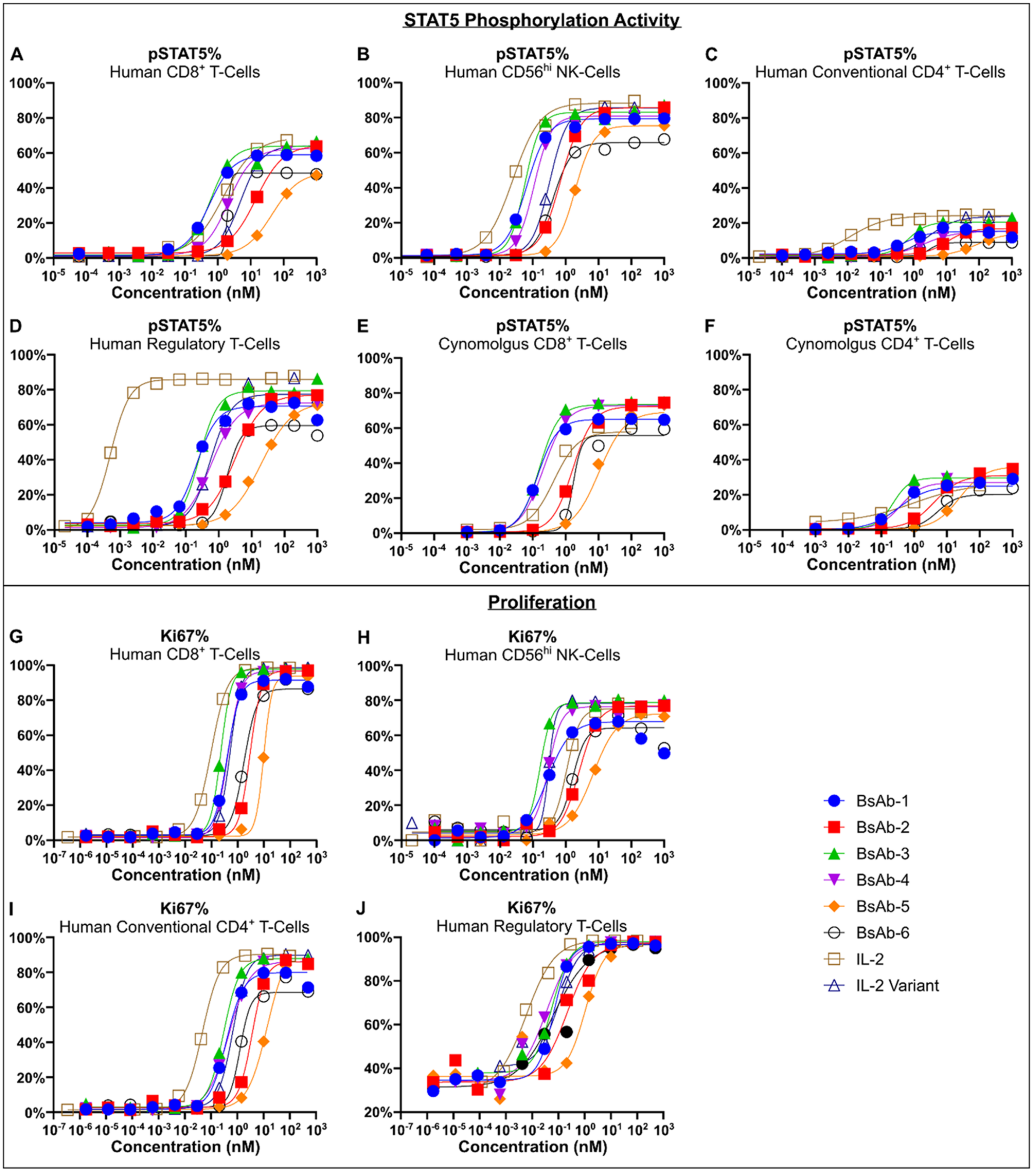
IL-2Rβγ bispecific UniAbs induce proliferation and STAT5 phosphorylation in PBMCs. (**A** to **F**) pSTAT5 dose curves for human CD8^+^ T-cells (**A**), CD56^hi^ NK-cells (**B**), CD4^+^ Conventional T-cells (**C**), CD4^+^ Regulatory T-cells (**D**), cynomolgus CD8^+^ T-cells (**E**), and total CD4^+^ T-cells (**F**). pSTAT5 levels were determined by flow cytometry and were reported as a percentage of the indicated cell type. (**G** to **J**) Ki67 dose curves for human CD8^+^ T-cells (**G**), CD56^hi^ NK-cells (**H**), CD4^+^ Conventional T-cells (**I**), CD4^+^ Regulatory T-cells (**J**). Ki67 levels were determined by flow cytometry and were reported as a percentage of the indicated cell type. CD4^+^ Conventional T-cells are defined here as CD3^+^CD4^+^Foxp3^-^. CD4^+^ Regulatory T-cells are defined here are CD3^+^CD4^+^CD25^+^Foxp3^+^.

The ability of the 6 IL-2Rβγ bispecific UniAbs to stimulate IL-2R signaling in human conventional CD4^+^ T, CD8^+^ T, and NK-cells was confirmed by a dose-dependent increase of STAT5 phosphorylation (Fig. 2A-C and Fig. S2A). The bispecific UniAbs were compared to two positive controls, rhIL-2 and an rhIL-2 variant which contains mutations (F42A, Y45A, L72G) that have been shown to disrupt binding to IL-2Rα while retaining the ability to bind and activate the intermediate affinity IL-2Rβγ receptor^28^. On CD8^+^ T-cells, the bispecific UniAbs exhibit a range of EC50 values in the pSTAT5 assay, with multiple leads (BsAb-1, BsAb-3, BsAb-4) showing near equivalent activity with rhIL-2 and the rhIL-2 variant (Fig. 2A). However, this is in stark contrast to the level of pSTAT5 in CD4^+^ CD25^+^ FoxP3^+^ T-regulatory cells, where the bispecific UniAbs show significantly lower potency compared to rhIL-2 on cells that express high levels of IL-2Rα (Fig. 2D). Thus, IL-2Rβγ bispecific UniAbs avoid the preferential activation of T-regs, as they bind the dimeric and trimeric IL-2 receptors equivalently, unlike native IL-2 which binds to the IL-2Rαβγ receptor at an affinity 100-fold higher than binding to IL-2Rβγ alone. All 6 bispecific UniAbs were also confirmed to activate IL-2R signaling on cynomolgus CD8^+^ and CD4^+^ T-cells, establishing cynomolgus monkeys as a suitable non-human primate model in subsequent studies (Fig. 2E,F).

To further compare the functional activity among the 6 bispecific UniAbs and rhIL-2, a cell proliferation assay was performed. In response to treatment with the bispecific IL-2R agonist UniAbs, or the IL-2 cytokine controls, immune effector cells (T and NK-cells derived from healthy donor PBMCs) demonstrated dose dependent proliferation (Fig. 2G-J and Fig. S2B). While a range of potencies was observed in the proliferation of CD8^+^ T-cells and NK-cells in PBMCs treated with the IL-2Rβγ bispecific UniAbs, several (BsAb-1, BsAb-3, BsAb-4) showed induction of proliferation at levels similar to rhIL-2 and the rhIL-2 variant control, while all molecules achieved similar levels of maximum proliferation (Fig. 2G,H). In contrast, rhIL-2 was more active than the bispecific agonist UniAbs and the rhIL-2 variant control on conventional CD4^+^ cells and on T-regs (Fig. 2I,J).

**Figure 3:**
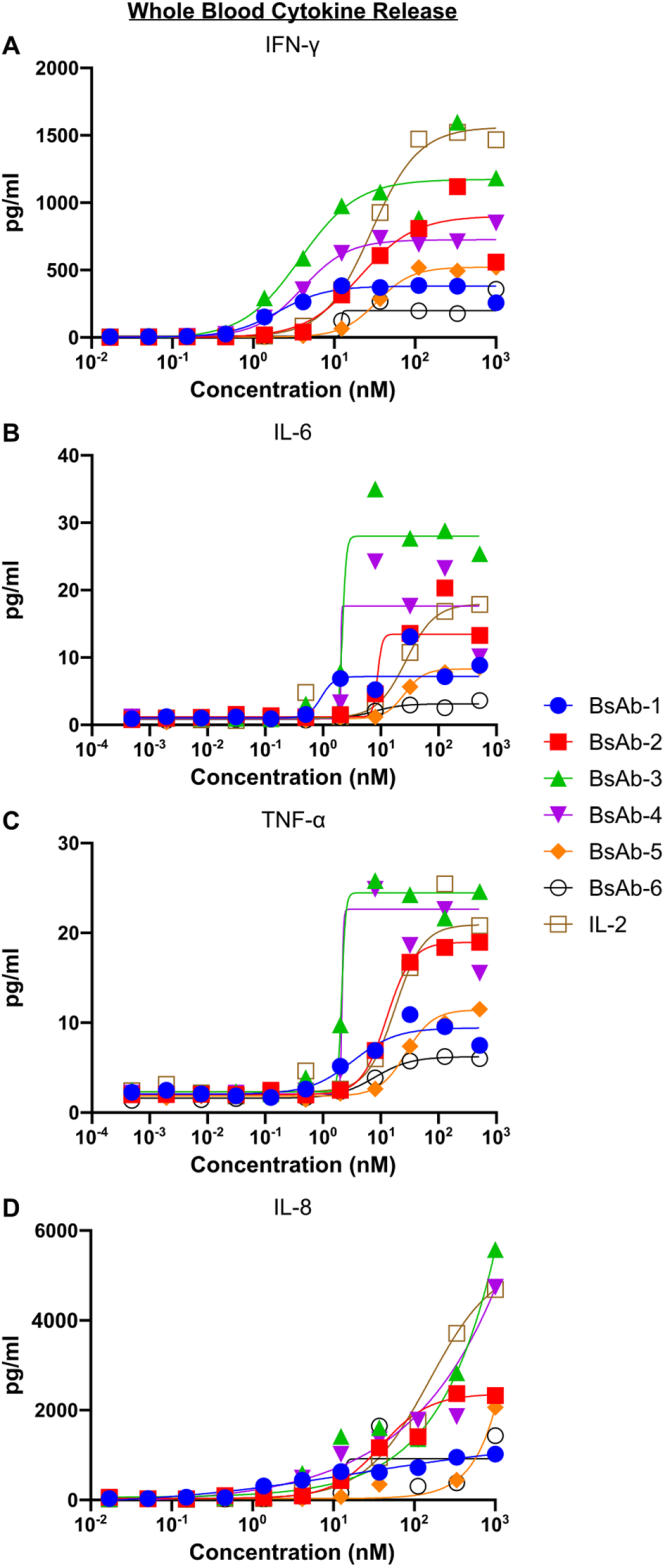
IL-2Rβγ bispecifics induce cytokine secretion in human whole blood. (**A** to **D**) Fresh human whole blood was incubated overnight with a range of doses of IL-2Rβγ bispecific UniAbs or IL-2. Plasma was then separated by centrifugation and tested by MSD for IFN-γ (**A**), TNF-α (**B**), IL-6 (**C**), and IL-8 (**D**).

Cytokine release profiles of the bispecific IL-2R agonist UniAbs compared to rhIL-2 were assessed in an ex vivo human whole blood assay. After a 24-h incubation in the presence of the IL-2Rβγ bispecific UniAbs or rhIL-2, a dose-dependent increase in IFN-γ, TNF-α, IL-6, and IL-8 was observed for all test articles (Fig. 3A-D). Two of the bispecific UniAbs (BsAb-3 and BsAb-4) induced cytokine levels (max concentration or EC50) at or above that of rhIL-2 in all tested cytokines, but the remaining four induced levels lower than the cytokine control. Notably, BsAb-1 and BsAb-2, displayed intermediate levels of cytokine production that were less than the highest levels of cytokine production elicited by the rhIL-2 control, but displayed greater than the lowest levels seen in BsAb-6.

In summary, 6 bispecific IL-2Rβγ antibodies were identified with a range of agonist activity. BsAb-1 demonstrates agonist activity at a similar level to that seen with rhIL-2 in immune effector cells measured by phosphorylation of STAT5 and in the proliferation assay. In contrast, in the same in vitro assays, BsAb-2 shows reduced potency compared to rhIL-2 and BsAb-1. Both antibodies showed low aggregation measured by SEC, had favorable melting temperatures, and were stable at 37 °C for one month (Table S1). These results combined with the favorable cytokine release profiles of BsAb-1 and BsAb-2 led to the selection of these two bispecific antibodies for further in vivo characterization. (Fig. 3).

### In vivo characterization of lead IL-2Rβγ bispecific UniAbs

**Figure 4:**
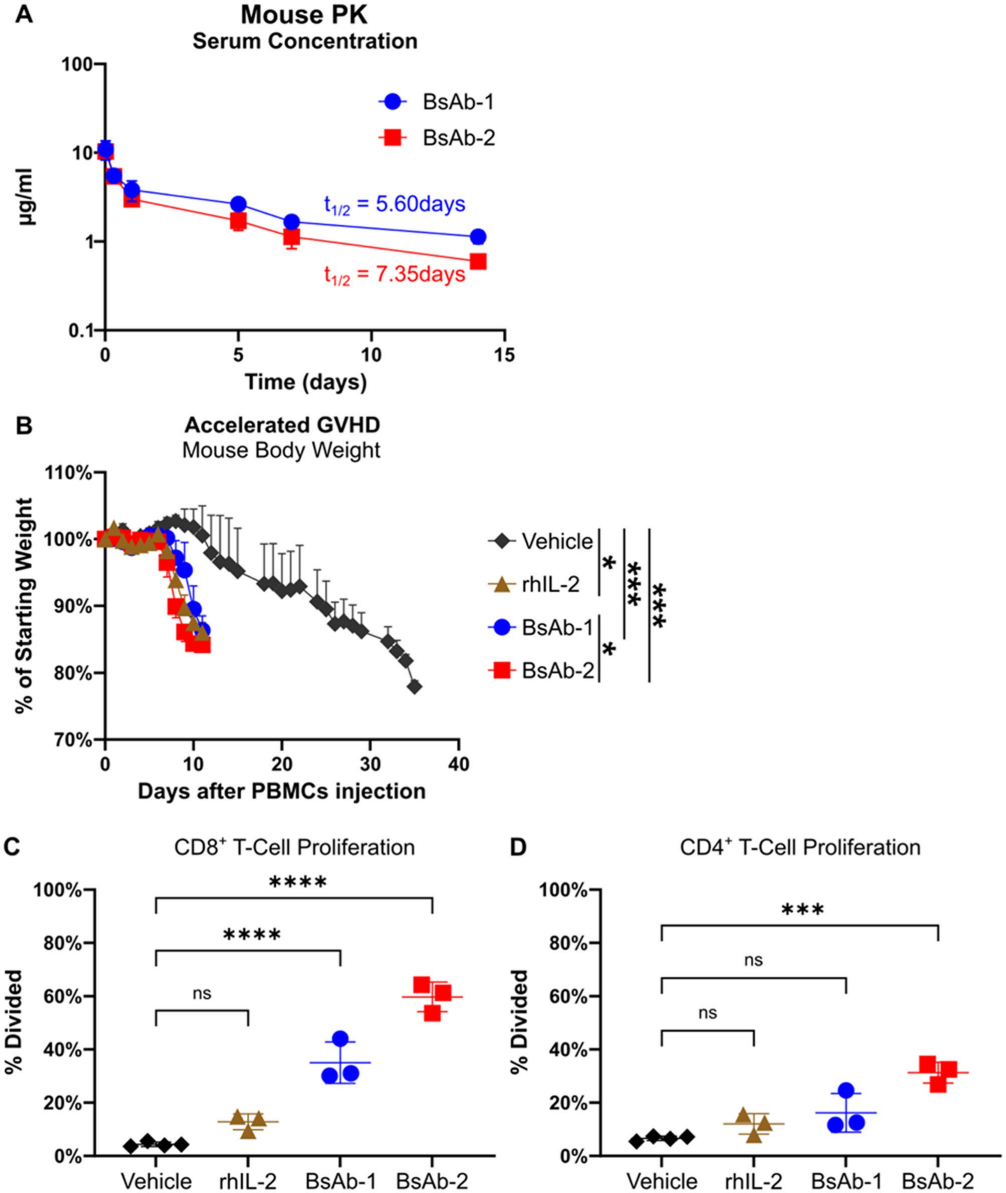
BsAb-1 and BsAb-2 induce T-cell proliferation and accelerated GVHD in a mouse model engrafted with human PBMCs. (**A**) BALB/c mice (n=3 per group per time point) were administered 1 mg/kg BsAb-1 or BsAb-2 by tail-vein injection. Serum was collected at 6 time points over two weeks and tested together by ELISA for human IgG4 using rhIL-2Rγ as a capture antigen. Error bars show SD. Pharmacokinetic parameters were determined using Phoenix pharmacokinetic software. (**B-D**) Irradiated NSG mice (5 per treatment group) were engrafted with 20 million human PBMCs each. Animals were then treated with either vehicle only, rhIL-2 (daily), or BsAb-1 or BsAb-2 (twice weekly) until ≥ 20% body weight loss. (**B**) Relative body weight of treated mice after injection of PBMCs. Horizontal lines indicate mean, and error bars show SEM. *p* values were calculated using a two-way RM ANOVA test. (**C** and **D**) Percentage of engrafted CD8^+^ T-cells (**C**) and CD4^+^ T-cells (**D**) which showed at least one round of division by CSFE staining. Representative histograms shown in Fig. S3. *p* values were calculated using a one-way ANOVA. Horizontal lines indicate mean, and error bars show SEM (**B**) or SD (**C** and **D**). *p < 0.05, **p < 0.01, ***p < 0.001, ****p < 0.0001.

Prior to conducting in vivo functional studies, the in vivo stability and pharmacokinetics of the bispecific antibodies were measured in mice. The observed 5–7 day half-life of each bispecific antibody is consistent with the half-life of a human IgG4 antibody in mice (Fig. 4A)^43^. To assess the in vivo functional activity of the bispecific antibodies, an accelerated graph versus host disease (GVHD) model was used to compare the functional activity of BsAb-1, BsAb-2 and rhIL-2 (Fig. 4B-D). In the first experiment, irradiated NSG mice were engrafted with human PBMCs, and the mice were subsequently treated with either vehicle, rhIL-2 daily, or one of the two bispecific agonist antibodies twice a week until sacrifice. As expected, animals treated with the vehicle control showed onset of GVHD, measured by body weight loss, around day 20 and were sacrificed with 20% body weight loss at approximately day 35. In contrast, the bispecific IL-2Rβγ agonist antibodies (BsAb-1, BsAb-2) as well as rhIL-2-treated animals exhibited onset of GVHD at approximately day 8 and were sacrificed with 20% body weight loss between days 9 and 13, indicating an acceleration of GVHD compared to the vehicle control, consistent with the enhanced activation of immune effector cells in treated mice (Fig. 4B).

A second study was conducted to directly measure the ability of BsAb-1 and BsAb-2 to stimulate the proliferation of immune effector cells in vivo. Similar to the first experiment, irradiated NSG mice were engrafted with human PBMCs that were labeled with CSFE and treated with vehicle, rhIL-2, BsAb-1, or BsAb-2. After day 5 of treatment, spleens were harvested and the proliferation of CD8^+^ T and CD4^+^ T-cells was compared between the 4 treatment groups by measuring CSFE staining in the different lymphocyte populations. BsAb-1 and BsAb-2 both showed significantly more proliferating CD8^+^ T-cells compared to rhIL-2 and the vehicle control (Fig. 4C, S3). CD4^+^ T-cells were expanded to a lesser extent; however, a significant increase in proliferating CD4^+^ T-cells was seen in BsAb-2 treated mice compared to the vehicle control (Fig. 4D).

**Figure 5:**
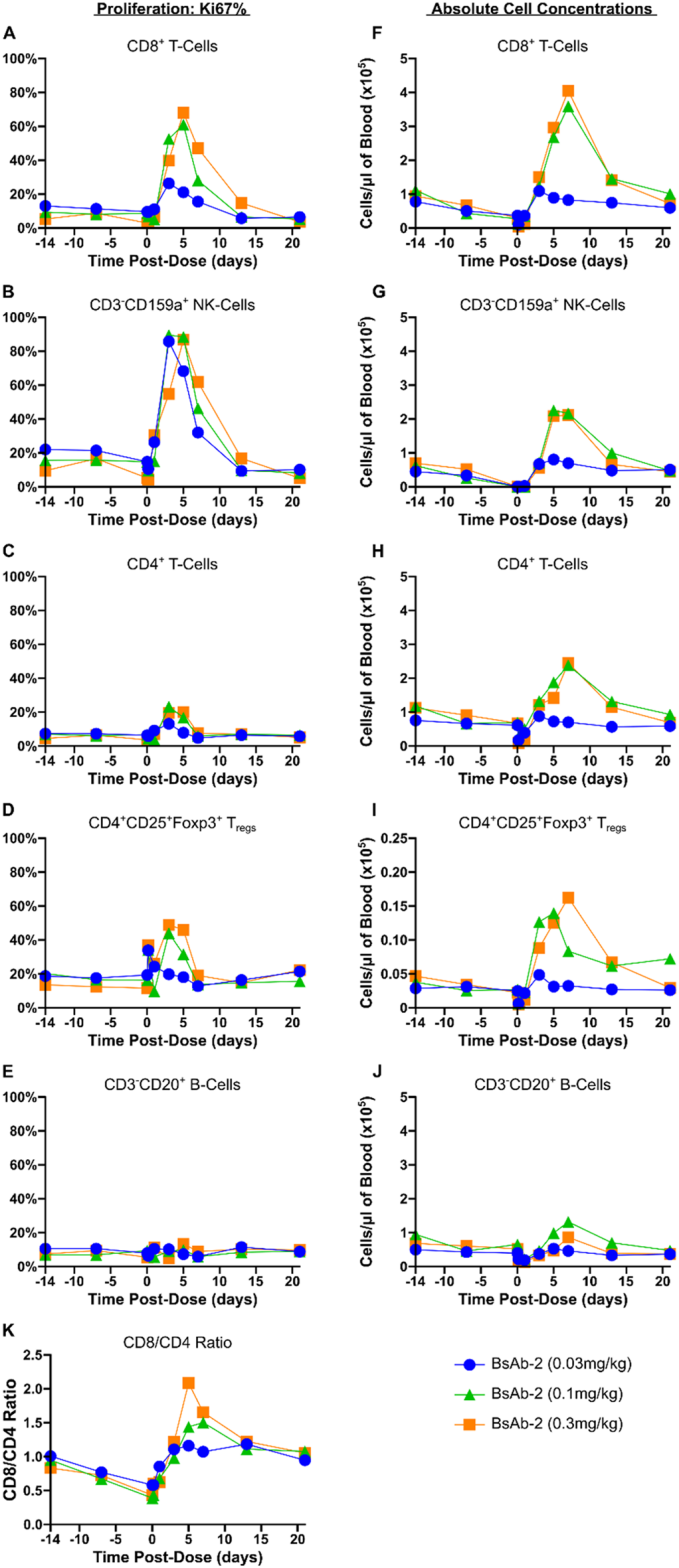
BsAb-2 induces dose-dependent lymphocyte proliferation in cynomolgus monkeys. Healthy cynomolgus monkeys (n = 2 per treatment group [1 male + 1 female]) were treated once with 0.03, 0.1, or 0.3 mg/kg body weight of BsAb-2 by intravenous injection. Peripheral blood was collected at 10 time points and cellular compartment was analyzed by flow cytometry. (**A** to **E**) Percentages of Ki67-expressing CD8^+^ T-cells (**A**), CD3^-^CD159a^+^ NK-cells (**B**), CD4^+^ T-cells (**C**), CD4^+^CD25^+^FoxP3^+^ T_regs_ (**D**), and CD3^-^CD20^+^ B-cells (**E**). (**F** to **J**) Absolute cell numbers of CD8^+^ T-cells (**F**), CD3^-^CD159a^+^ NK-cells (**G**), CD4^+^ T-cells (**H**), CD4^+^CD25^+^FoxP3^+^ T_regs_ (**I**), and CD3^-^CD20^+^ B-cells (**J**). Cell numbers determined using BD TruCount beads. (**K**) Ratio of CD8^+^ T-cells to CD4^+^ T-cells in cynomolgus peripheral blood.Data shown is the mean of the two subjects.

An important aspect of the preclinical evaluation of the bispecific antibody agonists was establishing cynomolgus monkeys as an appropriate in vivo model for measuring the pharmacodynamics of the molecules. To determine human and cynomolgus functional equivalency, the bispecific antibodies were confirmed to activate pSTAT5 signaling ex vivo in cynomolgus primary T-cells at a similar level (EC50s within tenfold) as seen in primary human T-cells (Fig. 2A,C,E,F). After establishing functional equivalency between human and cynomolgus, a cynomolgus study was conducted to further investigate the activity of BsAb-1 and BsAb-2 in vivo in a non-human primate model. The two bispecific agonist antibodies were administered to cynomolgus monkeys in groups of 2 that received a single intravenous (slow bolus) dose of 0.03, 0.1 or 0.3 mg/kg of either BsAb-1 or BsAb-2. At all doses with both lead molecules, a marked expansion of peripheral CD8^+^ T and NK-cells was observed (Fig. 5 and Fig. S4). After an initial transient drop in lymphocyte numbers, CD8^+^ T, NK-cells, and to a lesser degree, CD4^+^ T-cells, showed dose dependent proliferation and expansion in the blood, peaking around day 4–7 before returning to baseline levels around day 14 (Fig. 5A-C,F–H and Fig. S4A-C, F–H). Importantly, CD4^+^ CD25^+^ FoxP3^+^ T-regulatory cell expansion occurred at levels that were proportionally equivalent with the expansion of CD8^+^ T cells, consistent with the bispecific agonist antibodies avoiding preferential activation of the trimeric IL-2 receptor (Fig. 5D,I and Fig. S4D,I). The expansion of a overall higher number of CD8^+^ T-cells was further confirmed by the ratio of CD8^+^ :CD4^+^ T-cells which was skewed in favor of the CD8^+^ T-cell subset (Fig. 5K and Fig. S4K). Moreover, the IL-2Rβγ agonist antibodies were well tolerated in the monkeys at all dose levels tested, with no indication of vascular leak syndrome or other overt toxicities based on clinical observations. The Institutional Animal Care and Use Committee (IACUC) of Charles River Lab did not approve the use of rhIL-2 (proleukin) in this study due to the known severe toxicity of IL-2 in cynomolgus monkeys.

## Discussion

High-dose rhIL-2 (Proleukin^®^) is approved for the treatment of metastatic melanoma and renal cell carcinoma due to its anti-tumor efficacy in a subset of patients^8–11^. However, the effectiveness of high dose rhIL-2 is limited by its narrow therapeutic window due in part to the preferential activation and expansion of regulatory T-cells through the binding of the high-affinity IL-2 receptor composed of IL-2Rα, IL-2Rβ and IL-2Rγ subunits expressed on these cells. Adverse events, including vascular leak syndrome, further compromise its use in the clinic, limiting eligible patients and restricting its use to specialized treatment centers^6^. There has been substantial effort in the field to create mutations in the rhIL-2 protein which disrupt binding to the IL-2Rα subunit to avoid preferential binding to the high-affinity trimeric IL-2 receptor and overcome these unwanted side effects^24,25,28^. While achieving the desired function, these mutant proteins have also been shown to be immunogenic and must be further conjugated to a larger molecule to confer a longer in vivo half-life^30,31,44^.

In this study, we describe a novel approach for overcoming the limitations of rhIL-2 treatment by designing a fully human bispecific agonist antibody that activates immune effector cells through simultaneous binding and activation of the IL-2R beta and gamma subunits while avoiding binding to the IL-2R alpha subunit. Our motivation for taking this approach was primarily influenced by the favorable drug-like properties of an antibody format along with the exquisite specificity of bispecific antibodies. A bispecific antibody has the dual benefits of achieving the desired biological activity in a format with well-established manufacturing processes and superior drug-like properties. In support of these considerations, the IL-2Rβγ bispecific agonists we created can activate immune effector cells while avoiding preferential activation of T-regs. While the bispecific agonists maintain equivalent binding to IL-2Rβγ on T-regs and effector cells, the large excess of effector cells compared to T-regs means that many more effector cells are activated by the bispecific antibodies compared to T-regs. This is in contrast to native IL-2 which binds to IL-2Rαβγ on T-regs at a 100-fold higher affinity than IL-2Rβγ, thus biasing the activity of IL-2 to T-regs. The biological activity of the molecules, combined with Fc-mediated half-life extension, offers the opportunity to increase anti-tumor efficacy as well as reduce dose-limiting toxicities associated with rhIL-2 treatment.

Due to the pleiotropic nature of IL-2, there are benefits to considering alternative mechanisms of activating the IL-2 signaling pathway independent of IL-2, and IL-15 is one such alternative. While rhIL-15 has shown anti-tumor activity in clinical trials, it also exhibits considerable toxicity and a short serum half-life, similar to rhIL-2^45–47^. One of the challenges with the clinical application of rhIL-15 is that the IL-15Rα subunit is expressed in trans on antigen presenting cells. With this consideration, recent efforts have focused on developing a single chain construct that combines rhIL-15 with the IL-15 receptor αSu fragment to avoid the high affinity binding to the IL-15Rα chain on monocytes and dendritic cells^48–50^. The IL-2R agonist activity induced by the bispecific IL-2Rβγ antibodies is similar to IL-15-induced signaling as both bind to the beta and gamma subunits of the IL-2 receptor and avoid preferential activation of T-regulatory cells through interaction with IL-2Rα.

The expansion of T-cells along with an increased ratio of CD8^+^ T-cells to T-reg cells has been correlated with superior responses in patients^51–53^. In a mouse model engrafted with human PBMCs, the IL-2Rβγ agonist bispecific antibodies described in this paper stimulate the activation and expansion of T-cells equivalent to or better than that of rhIL-2. Furthermore, in cynomolgus monkeys, these bispecific antibodies are capable of inducing both T and NK-cell proliferation without a preferential expansion of T-reg cells reflected by a greater CD8 to CD4 ratio (see Fig. 5K and Fig. S4K). These in vivo observations of effector T-cell proliferation suggest that the IL-2R antibody agonists could facilitate a beneficial anti-tumor response in humans. However, one of the risks associated with T-cell proliferation and activation is the increased production of proinflammatory cytokines. To address this potentially negative side-effect of an IL-2R agonist, cytokine release was measured in a whole blood ex vivo study. In addition to stimulating immune cell proliferation, BsAb-1 and BsAb-2 also induced less proinflammatory cytokines than rhIL-2 in the whole blood cytokine release assay as shown in Fig. 3. Furthermore, both BsAb-1 and BsAb-2 were well tolerated in cynomolgus monkeys, where pro-inflammatory cytokines such as TNF-α and IL-6 were undetectable in the serum, and there were no clinical signs of cytokine release syndrome or vascular leak toxicity.

An IL-2Rβγ bispecific antibody agonist with an improved safety profile that expands immune cells in the tumor and periphery may be particularly effective when used in combination with other approved cancer therapeutics such as immune checkpoint inhibitors, ADCC competent monoclonal antibodies, and bispecific T-cell engagers. Checkpoint inhibitor antibodies and T-cell engaging bispecific antibodies both rely on a prevalent reservoir of healthy T-cells. Likewise, monoclonal antibodies that act through an ADCC mechanism rely on an ample population of NK-cells. Therefore, co-treatment with a bispecific IL-2Rβγ agonist that increases the abundance of these effector cells should benefit these types of therapies provided the toxicity of the combination treatment is tolerable. As an example of such a combination approach, NKTR-214/BEMPEG has been shown to be safe and has increased efficacy in combination with a checkpoint inhibitor in clinical trials^54^. The use of the ADCC competent monoclonal antibody, rituximab, in combination with the fusion protein L19-IL2 has also been shown to enhance activity compared to rituximab alone in B-cell lymphoma models^55^. While it has not been explored in the clinic yet, a third intriguing possibility would be to combine an IL-2Rβγ bispecific antibody with a next-generation T-cell engager exhibiting minimal cytokine release in order to increase the population of effector T-cells that can be recruited to the site of the tumor.

Other important considerations for an IL-2-based therapy is the localization and potency of the drug. There have been many efforts to conjugate IL-2 and other cytokines to monoclonal antibodies (immuno-cytokines) in an attempt to target the cytokine to the site of the tumor^28,56^. While this is a reasonable strategy to avoid off-tumor side effects, clinical benefit has been seen with multiple untargeted IL-2 variant molecules such as NKTR-214, THOR-707, and ALKS-4230^23,24,54,57^. While lacking tumor targeting, it is also interesting to note that BsAb-1 described in this study showed more potent in vitro activity compared to BsAb-2, but BsAb-2 had stronger in vivo effects compared to BsAb-1. This may be explained by BsAb-1 having a faster target-based clearance due to its higher binding affinity to the IL-2Rβγ receptor. Future studies will determine the pharmacokinetic properties of these bispecific antibodies in cynomolgus monkeys.

Taken together, the novel structure and mechanism of an IL-2Rβγ bispecific antibody agonist has many advantages for more fully exploiting the beneficial aspects of IL-2. Not only does the bispecific antibody achieve the desired biological activity, but it also has advantages of the well-established antibody format, and the approach we demonstrate in this study with IL-2R could be applied to other heterodimeric receptor signaling pathways. The in vitro and in vivo activity of the bispecific antibodies support the proposed biological mechanism that is consistent with other IL-2 variant proteins, and the preliminary results in cynomolgus monkeys provide evidence that this is a safe and well-tolerated molecule. Future studies will establish the maximum tolerated dose and pharmacokinetics in non-human primates with the ultimate goal of developing a safe and effective therapy for human cancer patients.

## Materials and methods

### Immunizations, next-generation sequencing, clonotype analysis and cloning

Methods essentially as described in Harris et al. *Front Immunol*. 2018 Apr 24; 9:889^34^. In brief, UniRat animals were immunized using standard adjuvants (Complete Freunds or Titermax/Ribi) along with recombinant protein antigens in a 48-day protocol or DNA immunizations. For protein immunizations, boosts consisted of 10 µg of recombinant protein injected into each leg of each animal with the appropriate adjuvant. In the case of DNA immunizations, gold particles were coated with vectors containing cDNA of the target antigen, which were subsequently administered subcutaneously every 7 days, using a gene gun. Plasma samples were collected post-immunization to assess serum titers against the antigen by ELISA.

After approximately 7 weeks (protein antigen) or 10 weeks (DNA antigen) of immunization, draining lymph nodes were harvested and total RNA was isolated. Ig heavy chain sequences were amplified using first strand cDNA synthesis and 5′ RACE by PCR, following methods similar to those previously described in Harris et al*. Front Immunol*. 2018 Apr 24; 9:889 and then purified by gel extraction^34^.

Next-generation sequencing was completed using the MiSeq platform (Illumina) with 2 × 300 paired-end reads. To enable multiplexing of samples, indexing labels were added by primer extension. Approximately 100,000 paired reads covered each sample, and those that showed alignment of less than 20 nucleotides to a human Ig locus were discarded. Merged forward and reverse reads of VH regions were translated into open reading frames and framework and CDR regions identified by IGBLAST (https://www.ncbi.nlm.nih.gov/igblast/). Clonotypes (defined by CDR3 protein sequences with at least 80% sequence similarity) were determined for samples using agglomerative clustering. CDR3 clonotypes were ranked by the percent of total reads in a sample defined by that clonotype. Those with the greatest abundance were prioritized for high-throughput cloning into an expression vector containing a CH1-deleted human IgG1 Fc region and validated by Sanger sequencing. Plasmids were transformed into *E. coli* grown in LB culture media and then purified to enable transient transfection of HEK 293 cells in 96-well format. Following several days of expression, supernatants containing antibody were harvested and clarified by centrifugation.

### High throughput ELISA

Methods essentially as described in Harris et al. *Front Immunol*. 2018 Apr 24; 9:889^34^. Briefly, recombinant proteins were coated overnight at 4 °C in 96-well plates using BupH Carbonate-Bicarbonate buffer (human IL-2Rβ, Acrobiosystems; cynomolgus IL-2Rβ, Sino Biological). Plates were then washed with TBST (20 mM Tris, 150 mM NaCl, 0.05% Tween-20, pH 7.6) and blocked with blocking buffer (TBST with 1% dry milk powder). HEK 293 supernatants containing antibodies were diluted 1:100 in blocking buffer and added to antigen-coated plates. Detection of bound antibodies was accomplished using an HRP-labeled anti-human Ig secondary antibody together with chemiluminescent substrate. Luminescence was quantified (SpectraMax i3X, Molecular devices) and the signal for each well was normalized by dividing by the average background luminescence of antigen-coated wells that had been incubated with supernatant from untransfected HEK 293 cells.

### Cell lines and PBMCs

M07e cells were obtained from DSMZ and were grown in RPMI medium containing 10% Fetal Bovine Serum (FBS), 1% Penicillin/Streptomycin, and 10 ng/mL rhGM-CSF. HSC-F cells were obtained from The Nonhuman Primate Reagent Resource and cultured in RPMI medium supplemented with 20% FBS, 1% Penicillin/Streptomycin and 55 μM β-Mercaptoethanol. 293-F were obtained from Gibco and grown according to their recommendations.

For creating stable cell lines expressing human IL-2Rβ or cynomolgus IL-2Rβ, expression constructs carried the full-length cDNA for the antigen and a Neo^R^ selection cassette. Each expression construct was then linearized and used to electroporate CHO cells. Three days after transfection, cells were put under selection for 3–6 weeks using Geneticin treatments. At the end of the selection period, all untransfected and negative control cell lines were killed, while all transfected pools showed re-growth as expected for successfully transfected pools. Four pools of each target were then assayed by flow cytometry for binding to a positive control antibody. The culture media for the CHO cells is as follows—EX-CELL® 325 PF CHO media containing 8 mM L-glutamine, 0.1 µg/L IGF-1, 5% dialyzed FBS, 0.45 mg/mL geneticin, and 0.45 mg/mL hygromycin. The cells were grown in suspension and maintained at a concentration between 0.5E6/mL to 2E6/mL.

Human PBMCs were isolated in-house from fresh leukapheresis packs (StemCell) by Ficoll® Paque Premium (GE Healthcare Life Sciences) density gradient centrifugation.

### Cell binding by flow cytometry

All washes and dilutions of cells, antibodies, and reagents were performed using flow buffer (1X PBS, 1% BSA, 0.1% NaN_3_, pH 7.4). Staining was performed in a round-bottom 96-well plate (Corning) seeded at 100,000 cells/well and all incubations were performed at 4 °C or on ice. For primary and secondary screens, the cells were incubated for 30 min with pre-diluted test antibodies (secondary screen and dose-curves) or 1:5 diluted HEK 293 supernatants containing antibodies (for primary screens and diversity screens) in a total volume of 50 μL. The cells were washed twice with 200 µL flow buffer. The cells were then incubated for 30 min with detection antibody (Goat F(ab')2 Anti-Human IgG-PE, Southern Biotech) at 0.625 µg/mL in flow buffer. Following 2 more washes, the cells were resuspended in a final volume of 150 µL of flow buffer. The cells were analyzed on a BD FACSCelesta or a Guava easyCyte 8-HT flow cytometer. At least 3000 events were collected, and PE geometric mean fluorescence intensity was plotted as a fold over background (cells incubated with secondary detection antibody only). In some secondary screens involving human or cynomolgus PBMCs, an additional CD4 antibody (BioLegend) and/or CD8 antibody (BioLegend) was included to further characterize cell binding.

### pSTAT5 detection by flow cytometry

For detection of pSTAT5 by flow cytometry, PBMCs were prepared from either frozen whole blood (cynomolgus) or frozen LeukoPak (human). Cells were thawed, washed twice with complete RPMI medium and resuspended at 5e6 cells/mL. 100 µL/well of these cells was then transferred to a sterile, round-bottom, 96-well plate (Corning) and sealed with an AeraSeal™ (Excel Scientific). The plate was then incubated at 37 °C and 5% CO2 for 1 h. After the incubation, 100 µL of pre-diluted antibodies (or IL-2 / IL-2 variant) was added to the appropriate wells. A final concentration of 10 nM IL-2 (R&D Systems) was used in control wells to ensure detectable pSTAT5. The plate was then resealed and returned to the incubator for an additional 1 h. After the incubation, the cells were centrifuged and washed twice with PBS pre-chilled to 4 °C. The cells were then blocked with Human TruStain FcX (BioLegend) and then subsequently stained for 30 min with Fixable Viability Dye (Invitrogen) and antibodies against CD3, CD4, CD8, CD25, and/or CD56. After staining, the cells were again centrifuged and washed twice with pre-chilled PBS. The cells were then fixed with the addition of 200 µL/well Fixation Buffer (BioLegend) and incubated at room temp for 30 min. After fixation, the cells were centrifuged and washed twice with Flow Buffer (1X PBS, 1% BSA, 0.1% NaN3, pH 7.4). Next, the cells were permeabilized by resuspending in 200 µL/well True-Phos buffer (BioLegend) pre-chilled to -20 °C and transferred to a -20 °C freezer overnight. The following morning, the cells were centrifuged, washed twice with flow buffer, and subsequently stained for 30 min with anti-pSTAT5 (BD Biosciences). After two additional washes, the cells were resuspended in 125 µL/well flow buffer and acquired on a BD FACSCelesta.

### Ki67 detection by flow cytometry

For detection of Ki67 by flow cytometry, frozen human PBMCs (previously isolated in-house from a LeukoPak) were thawed and rested overnight in complete RPMI medium at 1e6 cells/mL. The morning of the assay, the PBMCs were washed with complete RPMI and resuspended at 1e6 cells/mL. Then, to each well of a sterile 96-well plate, 100 µL of PBMCs, 50 µL of 0.16× ImmunoCult (StemCellTech), and 50 µL of diluted antibody or rhIL-2 (R&D Systems) was added. 0.5× ImmunoCult was used for staining controls to ensure detectable Ki67 and CD25 signal for compensation. The plate was then covered and incubated at 37 °C and 5% CO_2_. After 3 days, the media was refreshed with 100 µL/well of the corresponding concentration antibody and ImmunoCult and then returned to the incubator. After 3 more days (6 days total), the cells were centrifuged and washed twice with PBS pre-chilled to 4 °C. The cells were then blocked with Human TruStain FcX (BioLegend) and then subsequently stained for 30 min with Fixable Viability Dye (Invitrogen) and antibodies against CD3, CD4, CD8, CD25, and/or CD56. After staining, the cells were again centrifuged and washed twice with pre-chilled PBS. The cells were then fixed and permeabilized for 1 h with 200 µL/well FoxP3/Transcription Factor Staining Buffer working solution (Invitrogen). After permeabilization, the cells were centrifuged, washed twice with permeabilization buffer, and subsequently stained for 30 min with anti-FoxP3 (BioLegend) and anti-Ki67 (BioLegend). After two additional washes, the cells were resuspended in 125 µL/well flow buffer and acquired on a BD FACSCelesta.

### Whole blood cytokine release assay

Cytokine secretion was detected using fresh human whole blood (heparinized) obtained from AllCells. The following method was adapted from B. Wolf et al. / Cytokine 60 (2012) 828–837^58^. 12.5 µL of 20X concentrated (diluted in 1X PBS) test article was added to each well of a sterile 96-well round bottom plate. To this, 237.5 µL of fresh, human whole blood was added to each well with minimal pipetting to reduce non-specific activation. The plate was the covered and incubated at 37 °C and 5% CO2 overnight. The following morning, the plate was centrifugated at 1800 × g for 10 min and then 50 µL of serum was transferred to a 96-well microplate. The serum was then immediately tested by MSD (#K15010K-1 or a custom U-Plex plate) or frozen at − 80 °C for later testing.

### Mouse pharmacokinetic (PK) evaluation

The PK of BsAb-1 and BsAb-2 were each evaluated in 6 male BALB/c mice following a single tail vein injection of 1 mg/kg (n = 3 * 6 groups, Aragen Biosciences, Morgan Hill, CA). Serum samples were collected at selected time points over the course of 14 days post-dose.

### Mouse Accelerated GVHD Study

Each immune-compromised NSG mouse (8–9 weeks old from Charles River, France) was irradiated with 1.5 Gy on study day − 1. Mice were divided into 4 groups (n = 5) and 2 independent experiments were conducted using 2 different PBMC donors. On study day 0, each mouse was adoptively transferred IV with 20 million human PBMCs from one of the 2 donors and each mouse was treated with either vehicle control (100 µL), 22 µg rhIL-2 (350,000 IU/mice, Proleukin, Novartis) daily, 1 mg/kg BsAb-1 twice a week or 1 mg/kg BsAb-2 twice a week. GVHD was assessed by measuring weight loss over time in all animals. Animals were euthanized when body weight loss of 20% was observed.

In the second experiment, NSG mice (8–9 weeks old from Charles River, France) were irradiated with 1.5 Gy on study day -1. Mice were divided into 4 groups and 2 independent experiments were conducted using 2 different PBMC donors. On study day 0, each mouse was adoptively transferred IV with 20 million CSFE-labelled human PBMCs from one of the 2 donors and each mouse was treated with either vehicle control (100 µL) (n = 7), 22 µg rhIL-2 (350,000 IU/mice) daily (n = 6), 1 mg/kg BsAb-1 twice a week (n = 6) or 1 mg/kg BsAb-2 twice a week (n = 6). All animals were sacrificed on study day 5.

Immunophenotyping of the engrafted PBMCs by flow cytometry was performed on single-cell suspensions prepared from the mouse spleen on the day of sacrifice. The method of detection was largely the same as the above method for Ki67 detection by flow cytometry, but with different panels of antibodies to better distinguish the human PBMCs from the host cells. Cells were surface stained with anti-human CD45, CD3, CD4, CD8, CD25, CD16, CD19, and/or CD69. Following fixation and permeabilization, some of the cells were stained with anti-human FoxP3. The samples were then collected on a flow cytometer and analyzed using FlowJo analysis software.

### Cynomolgus pharmacodynamic (PD) study

The PD profiles of BsAb-1 and BsAb-2 were evaluated in twelve 2–4 years old naïve cynomolgus monkeys following a single IV (slow bolus) dose of 0.03, 0.1 or 0.3 mg/kg. Each treatment group contained 1 male and 1 female cynomolgus monkey (Charles River Lab, USA, Reno, NV). Blood samples were collected at selected time points for 21 days after dosing for analyses of hematology, serum chemistry, cytokines, and PD endpoints. After study termination, animals from the study were returned to the general colony. All procedures were approved by CRL IACUC and were performed in compliance with the Animal Welfare Act, the Guide for Care and Use of Laboratory Animals and the Office of Laboratory Animal Welfare.

### Cynomolgus blood immunophenotyping

A portion of the blood from each collected time point was used for immunophenotyping and quantification by flow cytometry. The method for Ki67 detection by flow cytometry was largely the same as described above, but with a different panel of cyno-reactive antibodies. Cells were surface stained with antibodies against CD3, CD4, CD8, CD20, CD25, and CD159a. After fixation and permeabilization, the cells were stained with antibodies against FoxP3 and Ki67. The samples were then collected on a flow cytometer and analyzed using FlowJo analysis software.

Simultaneously, a portion of each blood sample was transferred to BD TruCount tubes and stained with CD45 for real time quantification of peripheral blood cell absolute counts. The cell subset percentages from the above blood analysis were applied to the total cell numbers from the corresponding TruCount tube.

### αIgG4 ELISA

Serum concentrations of BsAb-1 and BsAb-2 in mouse serum were determined using an antigen capture ELISA. All washes and dilutions were performed with freshly made TBS-T (Accuris). All volumes should be assumed to be 100 µL/well except for coating, blocking and washing, which are at 200 µL/well. The night before the assay, Nunc MaxiSorp™ flat-bottom plates (Invitrogen) were coated with recombinant human IL2Rγ protein diluted to 1 µg/mL in carbonate-bicarbonate buffer (Thermo Scientific) and left at 4 °C. The next day, the plates were washed 5 times and then blocked with 1% BSA for 30 min. The plates were washed once and then multiple dilutions of the serum samples were added, along with a reference standard. Stocks of known concentration for BsAb-1 and BsAb-2 were used to make the standard curve. After 1 h at room temp, the plate was washed 8 times and then biotinylated anti-human IgG4-Fc (MABTECH) diluted to 3 µg/mL was added. The plates were incubated at room temp for another 30 min and then washed again 8 times. Next, the plates were incubated for 30 min with HRP-Streptavidin (Thermo Scientific) diluted 1:4000. Following an additional 8 washes, the plates were incubated in the dark for 6 min with room-temperature 1-Step Ultra TMB (Thermo Scientific). The reaction was stopped with 100 µL/well 2 N sulfuric acid. Absorbance was assessed at 450 nm and 570 nm.

### Protein expression and purification

Monospecific UniAbs were expressed in ExpiCHO cells following the manufacturer’s instructions (ThermoFisher A29133, Standard Protocol). Clarified supernatants were harvested on day 7 and purified using Protein A magnetic beads, using the KingFisher Flex Platform (ThermoFisher). Antibodies were eluted in 0.1 M citrate, 0.1 M NaCl, 10% glycerol, 10% sucrose, pH 3.5.

To express bispecific UniAbs, ExpiCHO cells were transfected with two expression vectors (knob and hole vectors, knob vectors contain C-terminal His-tag) and were expressed in the ExpiCHO cells according to manufacturer’s instructions using the high titer protocol. Clarified supernatants were harvested and the antibodies were purified by IMAC (Ni Sepharose® Excel, Cytive Life Sciences), using an imidazole gradient for elution. The IL-2Rβγ bispecific UniAbs containing fractions were pooled, concentrated, and further purified on cation exchange to remove any product-related impurities (Mono S® 10/100 GL column (Cytiva Life Sciences)). All antibodies were analyzed by SEC-UPLC and SDS-PAGE to confirm their size and purity.

The cynomolgus IL-2Rγ sequence was obtained from Uniprot.org (UniProt Accession ID: G7Q2Z6,) and the extracellular domain (aa Met1-Asn254) was cloned into a proprietary vector containing the endogenous leader sequence and a C-terminal His-tag. The IL-2Rγ reagent was expressed in ExpiCHO cells, according to the vendors instructions (high titer protocol, ThermoFisher). Cells were harvested on day 8 and supernatant was run on SDS-PAGE (NuPAGE 4–12% Bis Tris Gel) to verify target protein expression. Clarified harvest was purified by IMAC using Ni-Sepharose Excel resin (Cytiva Life Sciences), using an imidazole gradient for elution. The peaks were pooled and quantified using QiaXpert (Qiagen).

The cloning, expression, and purification of mutant IL-2 protein (T3A, F42A, Y45A, L72G, C125A) was completed at Lake Pharma. A C-terminal His-tag was added to enable purification by IMAC using standard procedures and elution with an imidazole gradient.

### Octet-based off-rate measurements

All off-rate measurements were performed on an Octet Qk384 instrument (ForteBio), in 96-well microplates at 25 °C using anti-human IgG Fc capture (AHC, 18–5005) sensors with a shake speed of 1000 rpm. For off-rate determination, the antibodies were loaded on the AHC sensors at 5 μg/mL. Following a short baseline in kinetics buffer (0.02% Tween20, 0.1% BSA, 0.05% sodium azide, 1× PBS). Offrate measurements were done for the following: human IL-2Rβ (AcroBiosystems), human IL-2Rγ (Sino Biological), cynomolgus IL-2Rβ (Sino Biological), cynomolgus IL-2Rγ (expressed and purified in house using ExpiCHO expression system followed by Ni–NTA His-tag purification), mouse IL-2Rγ (Sino Biological), mouse IL-2Rβ (Sino Biological), human IL-2Rα (Sino Biological), IL-4R (Sino Biological), IL-7R (Sino Biological), IL-9R (R&D Systems) and IL-21R (Sino Biological). The following antibodies were used as positive controls to verify target binding and reagent quality: anti-human IL-9R (R&D Systems), anti-human IL-21R (R&D Systems), anti-human IL-7R (R&D Systems) and anti-human IL-4Ra (R&D Systems). The loaded sensors were then submerged in wells containing antigen at 100 nM concentration for association step. Dissociation was monitored in kinetics buffer. The capture surfaces were regenerated for 60 s. ForteBio data analysis software was used to fit the data to a 1:1 binding model to extract an association rate and dissociation rate.

### Octet-based kinetics measurements

All kinetics measurement experiments were performed on a ForteBio Octet Qk384 instrument using anti-human Fc capture (AHC, 18–5005) sensors. The bispecific UniAbs and antigens were diluted to final concentrations in Kinetics buffer (0.02% Tween20, 0.1% BSA, 0.05% sodium azide, 1X PBS). Kinetics measurements were against the following antigens: human IL-2RB (AcroBiosystems), human IL-2RG (AcroBiosystems), cynomolgus IL-2RB (Sino Biological), cynomolgus IL-2RG (expressed and purified in house using ExpiCHO expression system followed by Ni–NTA his-tag purification). The antibodies were loaded on the AHC sensors at 5 μg/mL for maximum loading. Following a short baseline in Kinetics buffer, the sensors were exposed to a series of analyte concentrations (7.8 nM to 500 nM) for association step and background subtraction was used to correct for sensor drifting. Dissociation was monitored in Kinetics buffer. The capture surfaces were regenerated for 60 s. All experiments were performed with shaking at 1000 rpm. ForteBio’s data analysis software was used to fit the data to a 1:1 binding model to extract an association rate and dissociation rate. The KD was calculated using the ratio kd/ka.

### Biophysical characterization assay (T_m_, T_agg_)

T_m_ and T_agg_ were measured on the UNcle platform. Briefly, 9 µL of each sample was loaded in duplicate in a Uni (UNcle cassette) and run with a thermal ramp from 20 °C to 70 °C at a constant rate of 1 °C/min. UNcle Analysis 3.1 software, was used to calculate the T_m_ of each sample using the first derivative of the barycentric mean (BCM) of the fluorescence intensity. The T_agg_ for each sample was calculated using the intensity of scattered light at 266 nm.

### Thermal stress and stability characterization

Bispecific UniAb leads were concentrated to 10 mg/mL in 20 mM citrate and 0.1 M NaCl pH 6.2. Presence of high and low molecular weight species (%HMW and %LMW) was determined before and after temperature stress for 1 month at 2–8 °C and 37 °C by SEC on an analytical ThermoFisher UltiMate™ 3000 UPLC.

### Ethics statement

PBMCs from healthy, deidentified donors were isolated from LRS filters purchased from the Stanford Blood Center (Palo Alto, CA). Human PBMCs were collected in accordance with scientific, ethical, and regulatory guidelines. Animal studies were carried out in accordance with the recommendations in the Guide for the Care and Use of Laboratory Animals of the National Institutes of Health. Rat maintenance and immunizations were carried out by certified animal facilities in the U.S. (Antibody Solutions, Sunnyvale, CA) and Germany (Aldevron and MfD Diagnostics GmbH, Freiburg, Germany) in accordance with national and international guidelines, with protocols reviewed by Institutional Animal Care and Use Committee (IACUC) boards in the U.S. and comparable government boards in Germany. Mouse and cynomolgus studies were performed by AAALAC accredited facilities following internal approval by their IACUC boards (Aragen Biosciences, Morgan Hill, CA; Charles River Labs, Reno, NV; INSERM, Nantes, France).

### ARRIVE compliance

All studies in this paper were carried out in compliance with the ARRIVE guidelines.

## Supplementary Information

Supplementary Information.

## References

[1] Rosenberg SA (2014). IL-2: The first effective immunotherapy for human cancer. J. Immunol..

[2] Lotze MT (1985). In vivo administration of purified human interleukin 2. II. Half life, immunologic effects, and expansion of peripheral lymphoid cells in vivo with recombinant IL 2. J. Immunol. Baltim. Md..

[3] Fontenot JD, Rasmussen JP, Gavin MA, Rudensky AY (2005). A function for interleukin 2 in Foxp3-expressing regulatory T cells. Nat. Immunol..

[4] Boyman O, Sprent J (2012). The role of interleukin-2 during homeostasis and activation of the immune system. Nat. Rev. Immunol..

[5] Waldmann TA (2006). The biology of interleukin-2 and interleukin-15: Implications for cancer therapy and vaccine design. Nat. Rev. Immunol..

[6] Choudhry H (2018). Prospects of IL-2 in cancer immunotherapy. Biomed. Res. Int..

[7] Feinerman O (2010). Single-cell quantification of IL-2 response by effector and regulatory T cells reveals critical plasticity in immune response. Mol. Syst. Biol..

[8] McDermott DF (2004). Randomized phase III trial of high-dose interleukin-2 versus subcutaneous interleukin-2 and interferon in patients with metastatic renal cell carcinoma. J. Clin. Oncol..

[9] Payne R (2014). Durable responses and reversible toxicity of high-dose interleukin-2 treatment of melanoma and renal cancer in a Community Hospital Biotherapy Program. J. Immunother Cancer.

[10] Atkins MB (1999). High-dose recombinant interleukin 2 therapy for patients with metastatic melanoma: Analysis of 270 patients treated between 1985 and 1993. J. Clin. Oncol..

[11] Rosenberg SA, Yang JC, White DE, Steinberg SM (1998). Durability of complete responses in patients with metastatic cancer treated with high-dose interleukin-2. Ann. Surg..

[12] Saadoun D (2011). Regulatory T-cell responses to low-dose interleukin-2 in HCV-induced vasculitis. New Engl. J. Med..

[13] Koreth J (2011). Interleukin-2 and regulatory T cells in graft-versus-host disease. New Engl. J. Med..

[14] Krieg C, Létourneau S, Pantaleo G, Boyman O (2010). Improved IL-2 immunotherapy by selective stimulation of IL-2 receptors on lymphocytes and endothelial cells. Proc. Natl. Acad. Sci..

[15] Schwartzentruber DJ (2011). Gp100 peptide vaccine and interleukin-2 in patients with advanced melanoma. New Engl. J. Med..

[16] Rezvani K (2006). High donor FOXP3-positive regulatory T-cell (Treg) content is associated with a low risk of GVHD following HLA-matched allogeneic SCT. Blood.

[17] Sim GC (2014). IL-2 therapy promotes suppressive ICOS+ Treg expansion in melanoma patients. J. Clin. Invest.

[18] Murer P, Neri D (2019). Antibody-cytokine fusion proteins: A novel class of biopharmaceuticals for the therapy of cancer and of chronic inflammation. New Biotechnol..

[19] Arenas-Ramirez N, Woytschak J, Boyman O (2015). Interleukin-2: Biology, design and application. Trends Immunol..

[20] Charych DH (2016). NKTR-214, an engineered cytokine with biased IL2 receptor binding, increased tumor exposure, and marked efficacy in mouse tumor models. Clin. Cancer Res.

[21] Arenas-Ramirez N (2016). Improved cancer immunotherapy by a CD25-mimobody conferring selectivity to human interleukin-2. Sci. Transl. Med..

[22] Levin AM (2012). Exploiting a natural conformational switch to engineer an interleukin-2 ‘superkine’. Nature.

[23] Janku F (2020). 20 Poster discussion preliminary results from an open-label, multicenter phase 1/2 dose escalation and expansion study of THOR-707, a novel not-Alpha IL-2, as a single agent in adult subjects with advanced or metastatic solid tumors. Eur. J. Cancer.

[24] Lopes JE (2020). ALKS 4230: A novel engineered IL-2 fusion protein with an improved cellular selectivity profile for cancer immunotherapy. J. Immunother. Cancer.

[25] Silva D-A (2019). De novo design of potent and selective mimics of IL-2 and IL-15. Nature.

[26] Bernett MJ (2018). Abstract 5565: Potency-reduced IL15/IL15Rα heterodimeric Fc-fusions display enhanced in vivo activity through increased exposure. Undefined.

[27] Schliemann C (2012). Targeting interleukin-2 to the neovasculature potentiates rituximab‘s activity against mantle cell lymphoma in mice. Blood.

[28] Klein C (2017). Cergutuzumab amunaleukin (CEA-IL2v), a CEA-targeted IL-2 variant-based immunocytokine for combination cancer immunotherapy: Overcoming limitations of aldesleukin and conventional IL-2-based immunocytokines. Oncoimmunology.

[29] Groot ASD, Scott DW (2007). Immunogenicity of protein therapeutics. Trends Immunol..

[30] Webb GM (2020). The human IL-15 superagonist N-803 promotes migration of virus-specific CD8^+^ T and NK cells to B cell follicles but does not reverse latency in ART-suppressed, SHIV-infected macaques. Plos Pathog..

[31] van Brummelen EMJ (2018). 89Zr-labeled CEA-targeted IL-2 variant immunocytokine in patients with solid tumors: CEA-mediated tumor accumulation and role of IL-2 receptor-binding. Oncotarget.

[32] Verhoef JJF, Carpenter JF, Anchordoquy TJ, Schellekens H (2014). Potential induction of anti-PEG antibodies and complement activation toward PEGylated therapeutics. Drug Discov. Today.

[33] Clarke SC (2019). Multispecific antibody development platform based on human heavy chain antibodies. Front. Immunol..

[34] Harris KE (2018). Sequence-based discovery demonstrates that fixed light chain human transgenic rats produce a diverse repertoire of antigen-specific antibodies. Front. Immunol..

[35] Rickert M, Wang X, Boulanger MJ, Goriatcheva N, Garcia KC (2005). The structure of interleukin-2 complexed with its alpha receptor. Science.

[36] Sim GC, Radvanyi L (2014). The IL-2 cytokine family in cancer immunotherapy. Cytokine Growth Factor Rev..

[37] Ridgway JBB, Presta LG, Carter P (1996). ‘Knobs-into-holes’ engineering of antibody CH3 domains for heavy chain heterodimerization. Protein Eng. Des. Sel..

[38] Canfield SM, Morrison SL (1991). The binding affinity of human IgG for its high affinity Fc receptor is determined by multiple amino acids in the CH2 domain and is modulated by the hinge region. J. Exp. Med..

[39] Xu D (2000). In vitro characterization of five humanized OKT3 effector function variant antibodies. Cell Immunol..

[40] Bloom JW, Madanat MS, Marriott D, Wong T, Chan S (1997). Intrachain disulfide bond in the core hinge region of human IgG4. Protein Sci..

[41] Reddy MP (2000). Elimination of Fc receptor-dependent effector functions of a modified igg4 monoclonal antibody to human CD4. J. Immunol..

[42] Merchant AM (1998). An efficient route to human bispecific IgG. Nat. Biotechnol..

[43] Deng R (2011). Projecting human pharmacokinetics of therapeutic antibodies from nonclinical data. MAbs.

[44] Administration., U.S.D.A. Proleukin (aldesleukin) approval label. (2012).

[45] Conlon KC (2019). IL15 by continuous intravenous infusion to adult patients with solid tumors in a phase I trial induced dramatic NK-cell subset expansion. Clin. Cancer Res..

[46] Conlon KC (2014). Redistribution, hyperproliferation, activation of natural killer cells and CD8 T Cells, and cytokine production during first-in-human clinical trial of recombinant human interleukin-15 in patients with cancer. J. Clin. Oncol..

[47] Miller, J. S. *et al.* A First-in-Human Phase 1 Study of Subcutaneous Outpatient Recombinant Human IL-15 (rhIL-15) in Adults with Advanced Solid Tumors. *Clin Cancer Res* **24**, clincanres.2451.2017 (2017). 10.1158/1078-0432.CCR-17-2451 PMC6741437 29203590

[48] Xu W (2013). Efficacy and mechanism-of-action of a novel superagonist interleukin-15: Interleukin-15 receptor αSu/Fc fusion complex in syngeneic murine models of multiple myeloma. Cancer Res..

[49] Mathios D (2016). Therapeutic administration of IL-15 superagonist complex ALT-803 leads to long-term survival and durable antitumor immune response in a murine glioblastoma model. Int. J. Cancer.

[50] Margolin, K. *et al.* Phase I Trial of ALT-803, a Novel Recombinant Interleukin-15 Complex, in Patients with Advanced Solid Tumors. *Clin. Cancer Res.* **24**, clincanres.0945.2018 (2018). 10.1158/1078-0432.CCR-18-0945 PMC6239933 30045932

[51] Preston CC (2013). The ratios of CD8^+^ T cells to CD4^+^CD25^+^ FOXP3^+^ and FOXP3- T cells correlate with poor clinical outcome in human serous ovarian cancer. Plos One.

[52] Starkebaum G, Loughran TP, Waters CA, Ruscetti FW (1991). Establishment of an IL-2 independent, human T-cell line possessing only the p70 IL-2 receptor. Int. J. Cancer.

[53] Simpson TR (2013). Fc-dependent depletion of tumor-infiltrating regulatory T cells co-defines the efficacy of anti–CTLA-4 therapy against melanomaIntratumoral T reg cell depletion by α–CTLA-4. J. Exp. Med..

[54] Diab A (2020). Bempegaldesleukin (NKTR-214) plus Nivolumab in patients with advanced solid tumors: Phase I dose-escalation study of safety, efficacy, and immune activation (PIVOT-02). Cancer Discov..

[55] Schliemann C (2009). Complete eradication of human B-cell lymphoma xenografts using rituximab in combination with the immunocytokine L19-IL2. Blood.

[56] Johannsen M (2010). The tumour-targeting human L19-IL2 immunocytokine: Preclinical safety studies, phase I clinical trial in patients with solid tumours and expansion into patients with advanced renal cell carcinoma. Eur. J. Cancer.

[57] Vaishampayan UN (2020). ALKS 4230 monotherapy and in combination with pembrolizumab (pembro) in patients (pts) with refractory solid tumours (ARTISTRY-1). Ann. Oncol..

[58] Wolf B (2012). A whole blood in vitro cytokine release assay with aqueous monoclonal antibody presentation for the prediction of therapeutic protein induced cytokine release syndrome in humans. Cytokine.

