# Supplementary material for: A bispecific antibody agonist of the IL-2 heterodimeric receptor preferentially promotes in vivo expansion of CD8 and NK cells
Source: Sci Rep. 2021 May 19;11:10592. doi: 10.1038/s41598-021-90096-8 (PMC8134639; doi:10.1038/s41598-021-90096-8)
Supplement: Supplementary file 1 — Supplementary Information. [file 41598_2021_90096_MOESM1_ESM.docx]

**A bispecific antibody agonist of the IL-2 heterodimeric receptor preferentially promotes *in vivo* expansion of CD8 and NK cells**

**Supplemental Data**


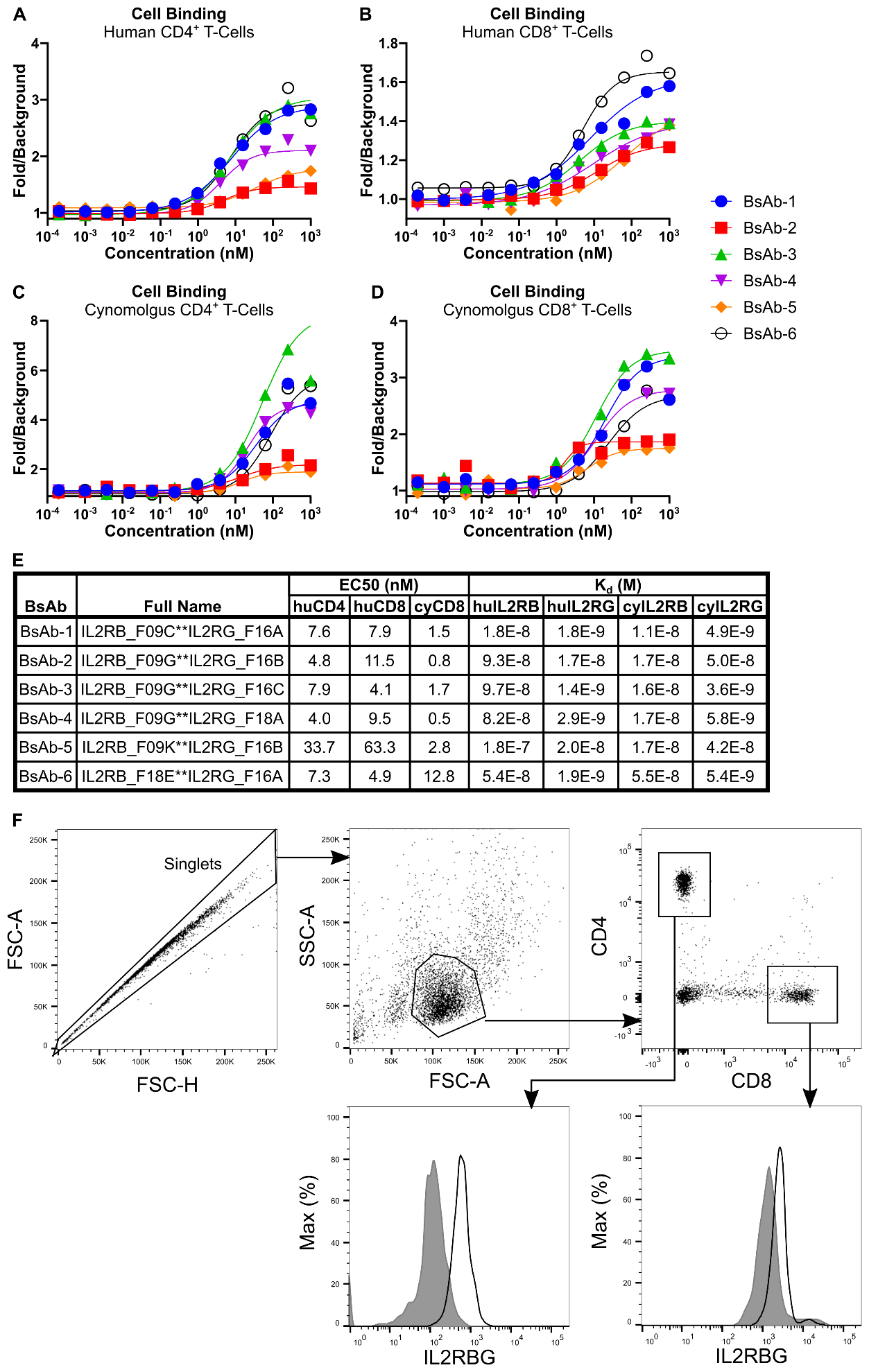


**Fig. S1. IL-2Rβγ bispecific UniAbs bind human and cynomolgus T-cells at comparable levels.** (A to D) Cell binding dose curves against human CD4+ T-cells (**A**) and CD8+ T-cells (**B**), or cynomolgus CD4+ T-cells (**C**) and CD8+ T-cells (**D**). Binding was determined by flow cytometry and reported as geometric mean fluorescent intensity (gMFI) over the gMFI of cells stained only with secondary detection antibody. (**E**) Summary table of the EC50s for the preceding graphs and binding affinities of bispecific UniAbs. EC50 values generated by GraphPad Prism. Dissociation constants (K_d_) of bispecific UniAbs for human and cynomolgus IL-2RB and IL-2RG were determined by global fitting of binding curves to 1:1 interaction model using Octet QK 384. (**F**) Representative gating strategy for determining CD4+ and CD8+ T-cell binding. The black outlined histogram shows the binding of the IL-2Rβγ bispecific antibody, while the gray filled-in histogram shows background fluorescence from the detection antibody when no IL-2Rβγ bispecific antibody is added.


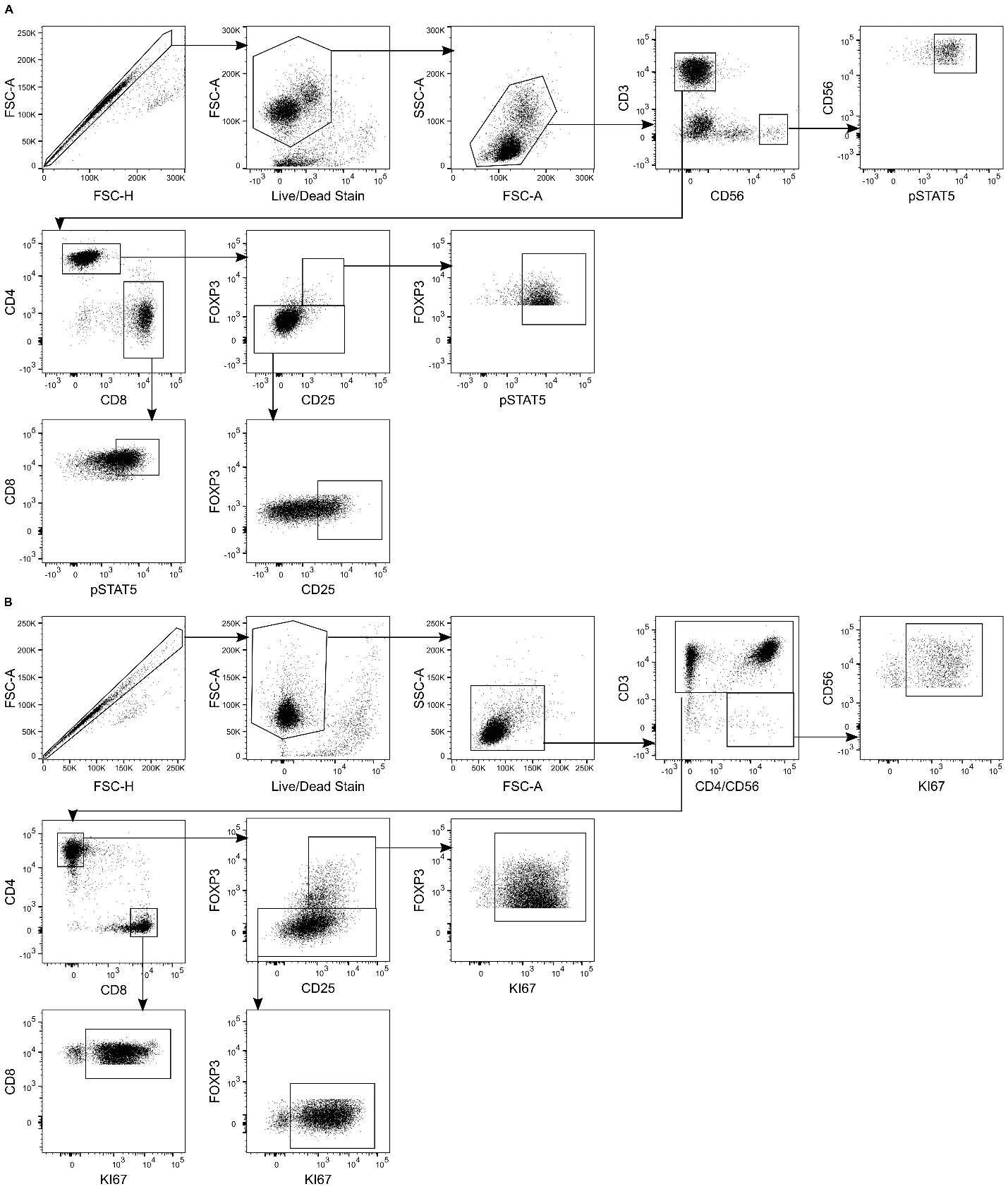


**Fig S2. Flow cytometry gating strategies.** Representative gating strategies for pSTAT5 (A) and Ki67 (B) assays shown in Fig 2.
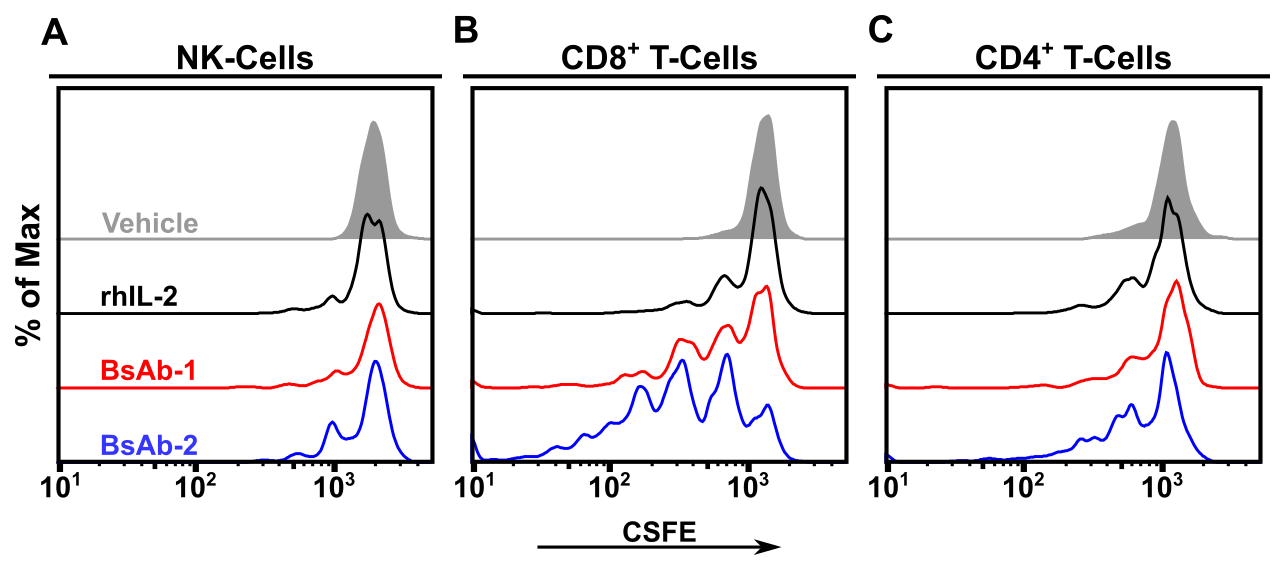


**Fig. S3. IL-2Rβγ bispecific UniAbs induce lymphocyte proliferation *in vivo*.** (**A** to **C**) Representative CSFE histograms showing the multiple cycles of division induced in NK-cells (**A**), CD8+ T-cells (**B**), and CD4+ T-cells (**C**) engrafted in irradiated NSG mice (n=5 per group) treated with either vehicle only, rhIL-2 (daily), or BsAb-1 or BsAb-2 (twice weekly).

**
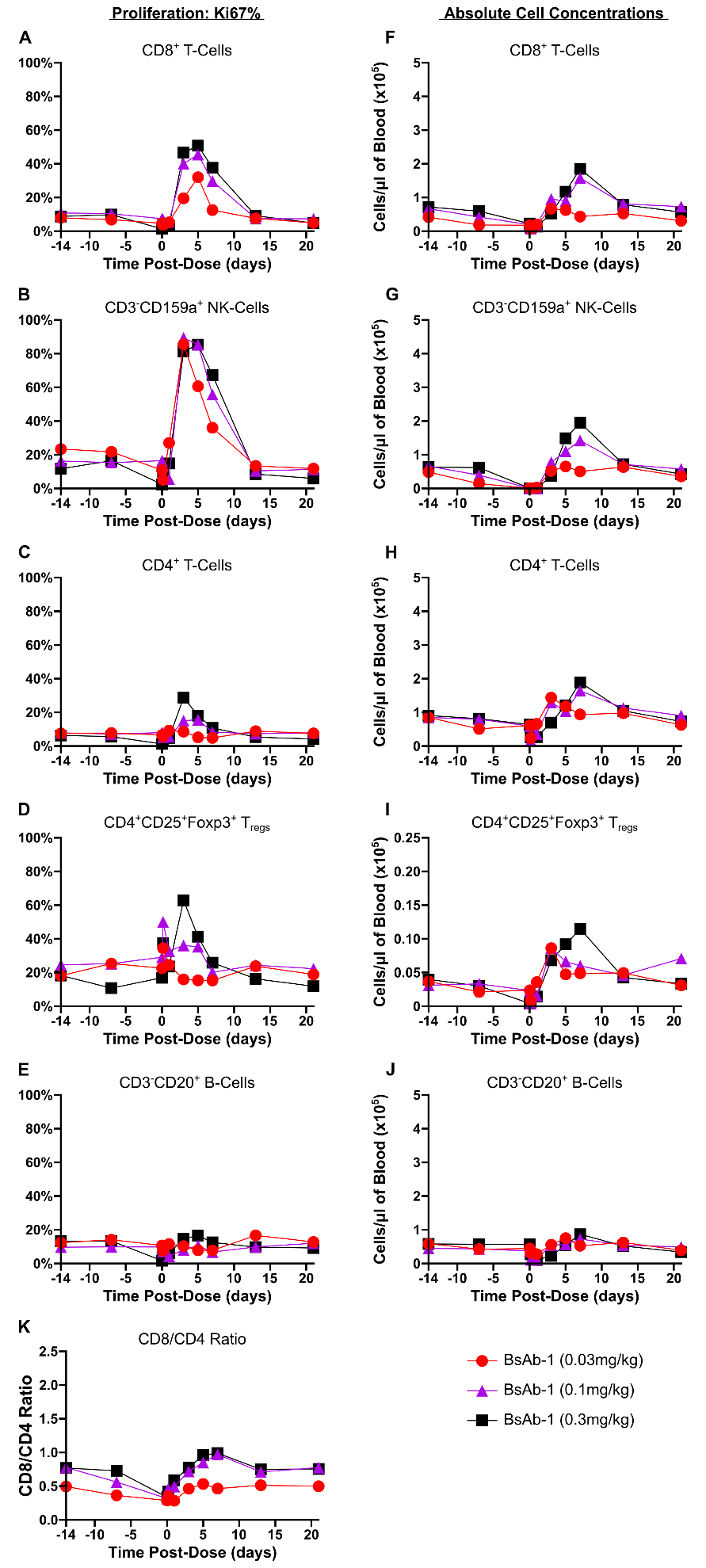
Fig S4. BsAb-1 induces dose-dependent lymphocyte proliferation in cynomolgus monkeys.** Healthy cynomolgus monkeys (n=2 per treatment group [1 male + 1 female]) were treated once with 0.03, 0.1, or 0.3 mg/kg body weight of BsAb-1 by intravenous injection. Peripheral blood was collected at 10 time points and cellular compartment was analyzed by flow cytometry. (**A** to **E**) Percentages of Ki67-expressing CD8^+^ T-cells (**A**), CD3^-^CD159a^+^ NK-cells (**B**), CD4^+^ T-cells (**C**), CD4^+^CD25^+^FoxP3^+^ T_regs_ (**D**), and CD3^-^CD20^+^ B-cells (**E**). (**F** to **J**) Absolute cell numbers of CD8^+^ T-cells (**F**), CD3^-^CD159a^+^ NK-cells (**G**), CD4^+^ T-cells (**H**), CD4^+^CD25^+^FoxP3^+^ T_regs_ (**I**), and CD3^-^CD20^+^ B-cells (**J**). Cell numbers determined using BD TruCount beads. (**K**) Ratio of CD8^+^ T-cells to CD4^+^ T-cells in cynomolgus peripheral blood. Data shown is the mean of the two subjects.


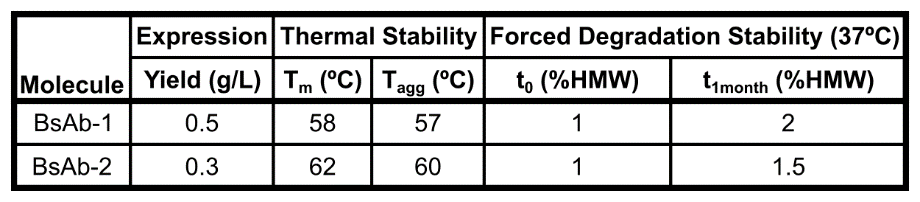


**Table S1. Bispecific UniAbs have favorable protein biophysical characteristics.** Summary of BsAb-1 and BsAb-2 properties. The expression yields of antibodies were determined post first capture step from a 200 mL supernatant. The leads were assessed for thermal stability by measuring the T_m_ and T_agg_. Additionally, the leads were assessed for forced degradation stability by measuring the percent high molecular weight (%HMW) species present by SEC post temperature stress.
